# Psychosis associated with sibutramine consumption: a case report

**DOI:** 10.1192/j.eurpsy.2026.11781

**Published:** 2026-07-31

**Authors:** A. Gomez Prieto, A. García Gonzalez, A. Mercado Rodriguez, I. Zorrilla Martinez, A. M. González-Pinto Arrillaga

**Affiliations:** Psychiatry, OSAKIDETZA, Vitoria, Spain

## Abstract

**Introduction:**

Sibutramine is a serotonin and noradrenaline reuptake inhibitor that was withdrawn from the market due to cardiovascular and neuropsychiatric adverse effects. Nevertheless, it continues to be clandestinely marketed in “natural” weight-loss products sold online, without proper labeling, thus posing a significant health risk. Psychotic episodes related to sibutramine intake have been reported, yet they are often underdiagnosed (1,2).

**Objectives:**

To describe a clinical case of psychosis secondary to sibutramine consumption, highlighting the recurrence of two clinically similar episodes after re-exposure and the relevance of a thorough clinical history, including over-the-counter supplements, in the differential diagnosis of first-episode psychosis.

**Methods:**

A 33-year-old woman, with no previous medical or psychiatric history, was involuntarily admitted after a six-month history of bizarre behavior, incoherent speech, persecutory and referential delusions, anxiety, and insomnia. Routine blood tests and urine toxicology were unremarkable. Brain MRI revealed an incidental Rathke’s cleft cyst.

**Results:**

During hospitalization, the patient disclosed chronic use of a slimming product (LiDa), later identified as adulterated with sibutramine. Following discontinuation of the product and initiation of risperidone 3 mg nightly, she showed rapid and complete remission. Two years later, after re-exposure to the same supplement, she developed a second psychotic episode with identical features, which again resolved with the same therapeutic approach.

**Conclusions:**

Sibutramine can trigger acute psychotic episodes in individuals without prior psychiatric history, with a risk of recurrence upon re-exposure. This case emphasizes the importance of a comprehensive clinical history exploring the use of supplements and “natural” products purchased online. Identifying such hidden agents as potential triggers is crucial to avoid underdiagnosis and prevent relapses.

**References:**

1. Chen SP, et al. Psychosis with herbal slimming products adulterated with sibutramine: a case series. Clin Toxicol (Phila) 2010;48:832–8.
2. Lee J, et al. Catatonia and psychosis associated with sibutramine: a case report. Prog Neuropsychopharmacol Biol Psychiatry 2007;31:1179–82.

**Disclosure of Interest:**

None Declared

